# SDOCT Imaging to Identify Macular Pathology in Patients Diagnosed with Diabetic Maculopathy by a Digital Photographic Retinal Screening Programme

**DOI:** 10.1371/journal.pone.0014811

**Published:** 2011-05-06

**Authors:** Sarah Mackenzie, Christian Schmermer, Amanda Charnley, Dawn Sim, Vikas Tah, Martin Dumskyj, Stephen Nussey, Catherine Egan

**Affiliations:** 1 Moorfields at St George's Hospital, London, United Kingdom; 2 Department of Endocrinology, St George's Hospital, London, United Kingdom; University of Cape Town, South Africa

## Abstract

**Introduction:**

Diabetic macular edema (DME) is an important cause of vision loss. England has a national systematic photographic retinal screening programme to identify patients with diabetic eye disease. Grading retinal photographs according to this national protocol identifies surrogate markers for DME. We audited a care pathway using a spectral-domain optical coherence tomography (SDOCT) clinic to identify macular pathology in this subset of patients.

**Methods:**

A prospective audit was performed of patients referred from screening with mild to moderate non-proliferative diabetic retinopathy (R1) and surrogate markers for diabetic macular edema (M1) attending an SDOCT clinic. The SDOCT images were graded by an ophthalmologist as SDOCT positive, borderline or negative. SDOCT positive patients were referred to the medical retina clinic. SDOCT negative and borderline patients were further reviewed in the SDOCT clinic in 6 months.

**Results:**

From a registered screening population of 17 551 patients with diabetes mellitus, 311 patients met the inclusion criteria between (March 2008 and September 2009). We analyzed images from 311 patients’ SDOCT clinic episodes. There were 131 SDOCT negative and 12 borderline patients booked for revisit in the OCT clinic. Twenty-four were referred back to photographic screening for a variety of reasons. A total of 144 were referred to ophthalmology with OCT evidence of definite macular pathology requiring review by an ophthalmologist.

**Discussion:**

This analysis shows that patients with diabetes, mild to moderate non-proliferative diabetic retinopathy (R1) and evidence of diabetic maculopathy on non-stereoscopic retinal photographs (M1) have a 42.1% chance of having no macular edema on SDOCT imaging as defined by standard OCT definitions of DME when graded by a retinal specialist. SDOCT imaging is a useful adjunct to colour fundus photography in screening for referable diabetic maculopathy in our screening population.

## Introduction

The U.K. diabetic population is predicted to increase by 1.5 fold by 2030.[1] Since 75% of diabetics have diabetic retinopathy 20 years after diagnosis, there is an increasing burden on medical retina clinics.[1], [2] Diabetic retinopathy screening (DRS) services have been established to reduce blindness due to sight threatening retinopathy. Diabetic macular edema (DME) is a common cause of sight-threatening retinopathy.[3] The Early Treatment Diabetic Retinopathy Study (ETDRS) identified patients with diabetic maculopathy at risk of vision loss. Macular laser was shown to prevent vision loss if applied according to protocol when the level of maculopathy reached so-called, ‘clinically significant macular edema’ (CSME). CSME was identified by the examination of stereoscopic fundus photographs, or a stereoscopic examination of the fundus and was independent of visual acuity. Patients with less severe levels of maculopathy, including so-called non-CSME levels of edema, did not achieve benefit from laser.[4]

Stereoscopic screening photographs are expensive and the inherent difficulty in diagnosing DME on retinal screening photographs which are not stereoscopic led to the identification of surrogate markers of DME, such as exudates and microaneurysms or haemorrhage in association with vision loss.[5] This results in referral of large numbers of patients, who do not have DME or CSME, and underdiagnosis of patients with DME and no retinal lesions or lesions with good visual acuity. Patients with a variety of other retinal lesions (microaneurysms, small retinal haemorrhages, exudates) and no evidence of macular edema or CSME suffer needless anxiety and confusion, when referred from community screening to hospital eye services.

There are multiple established international grading systems for diabetic retinopathy and maculopathy.[6], [7] The English National Screening Committee (ENSC) has introduced a simplified retinopathy grading system to facilitate population screening of the retina on a large scale.[6], [8] Patients graded as M1 are referred to ophthalmology clinics to be seen within 13 weeks (see Table 1 for ENSC grading).[8] A recently published audit of unselected referrals from another photographic screening service in England found that only 21% of patients graded as M1 by the screening programme were judged by the ophthalmologist in clinic to require laser treatment.[9]

**Table 1:** ENSC grading.

ENSC Grade		Clinical features
**Retinopathy Grade**	**R0**	No diabetic retinopathy
	**R1**	(Background) Microaneurysm(s), Retinal haemorrhage(s) ± any exudates (not within the definition of maculopathy)
	**R2**	(Pre-proliferative) venous beading venous loop or reduplication intra-retinal microvascular abnormality (IRMA) multiple deep, round or blot haemorrhages Cotton Wool Spots (careful search for above features)
	**R3**	(Proliferative) new vessels on disc (NVD) new vessels elsewhere (NVE) pre-retinal or vitreous haemorrhage pre-retinal fibrosis ± tractional retinal detachment
**Maculopathy Grade**	**M0**	No maculopathy
	**M1**	M1 grade is defined as: Exudate within 1 disc diameter (DD) of the centre of the fovea, or circinate or group of exudates within the macula, or retinal thickening within 1 DD of the centre of the fovea (if stereo available), or any microaneurysm or haemorrhage within 1 DD of the centre of the fovea only if associated with a best VA of (if no stereo) 6/12 or worse.
**Photocoagulation**	**P**	evidence of focal/grid laser to macula evidence of peripheral scatter laser
**Unclassifiable**	**U**	Unobtainable/un-gradable

DD; disc diameter, VA; visual acuity.

Optical Coherence Tomography (OCT) is an established, well-tolerated, non-invasive, non-contact method of imaging the macula.[10]–[14] It is a more accurate and objective method of diagnosing macular edema than clinical examination, even by experts.[15]–[20] Furthermore, OCT imaging is an established clinical trial endpoint for new treatments for DME. [21]–[24]

The spectral-domain OCT (SDOCT) machine used in this audit allows technical staff to generate both a high quality fundus photograph for retinal screening and a simultaneous OCT scan to diagnose macular edema in under one minute per eye.[25] There is the potential for longitudinal follow-up of individual patients owing to the high level of repeatability, the potential to train technical staff in grading principles and the opportunity for audit, telemedicine, and automation.[5], [18], [26]–[30]

We have analyzed the OCT images from patients graded as mild to moderate non-proliferative retinopathy with features on non-stereoscopic retinal photography of maculopathy (ENSC grade equivalent R1/M1) to find how many of these patients had no evidence of macular edema.

## Materials and Methods

A photographic diabetic retinal screening service (DRSS) based in the endocrinology department at St George's Hospital in South-West London has been in operation since 1999. This service participates in the English National Screening Programme and adheres to the national protocols for image capture, technician training, image grading, referral patterns and quality assurance. The service has 17,551 patients with diabetes on its fully collated list and uses the non-mydriatic fundus camera Topcon TRC-NW6S with a Nikon D80 camera body attached to it to collect fundus images after pupil dilation.[31] The protocol specifies 2 images per eye – one centred on the macula and the other on the optic nerve – with a field size of 45 degrees. All images are graded by trained staff according to National Screening Committee (NSC) protocols. Referral of M1 disease is to the ophthalmology clinic (Moorfields medical retina service) based on site.

**Figure 1:**
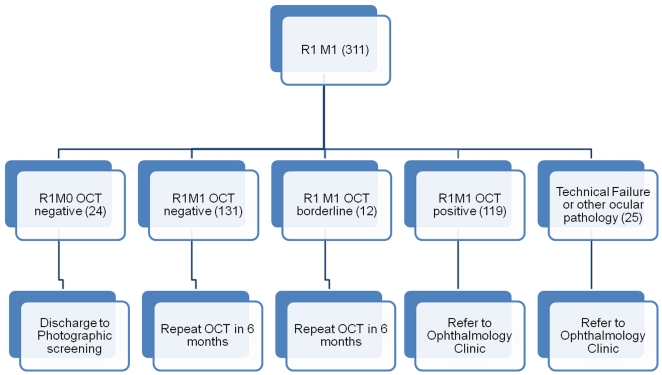
OCT clinic pathway. The number of patients identified in this study at each section of this pathway are highlighted. Note that any R2 and R3 identified at primary screening are referred directly to ophthalmology clinic and do not enter the SDOCT clinical pathway represented in Figure 1.

We report a prospective audit of patients referred from DRSS with R1/M1 maximal retinopathy in either eye attending an SDOCT clinic. We selected this group on the basis that the only reason for referral to an ophthalmologist would be the possible presence of DME. Patients with proliferative diabetic retinopathy, severe non-proliferative retinopathy or un-gradeable retinal images in either eye were excluded from this analysis and were referred in the usual way to ophthalmology clinics. We analyzed all consecutive imaging episodes in an SDOCT clinic between March 2008 and September 2009. Patients attending the SDOCT clinic had a visual acuity measurement, colour fundus photography and OCT scanning performed. A Topcon 3DOCT-1000 SDOCT was used with a 512 A scans x 128 lines raster scan protocol.[31] SDOCT scans were taken by trained technicians. All images were graded by a single consultant ophthalmologist specialising in medical retina as SDOCT positive (edema present), borderline or negative. OCT features used to grade scans as SDOCT positive were central subfield measurement ≥250 microns (a standard clinical trial entry criteria for DME), and additional criteria to include subjective features that may indicate DME at a very early stage, as follows: the presence of intra-retinal cysts, sub-retinal fluid and/or diffuse retinal edema (retinal thickening with areas of reduced retinal reflectivity) on more than one scan, or any of the above associated with a change in the internal limiting membrane (ILM) contour, including increased central macular thickness or loss of foveal contour. These guidelines include and extend those used in clinical trials [21]–[24], [32]. SDOCT positive patients were referred to the medical retina clinic according to the usual English national protocols and timelines. SDOCT borderline scans had an intra-retinal cystic space on a single scan without a change in the ILM contour. SDOCT borderline and SDOCT negative patients were booked for a further SDOCT clinic appointment in 6 months and images were again graded. OCT scans were also used to confirm the status of the original referral image as M1. For example, some patients have a pale central lesion that could be either exudate or a small drusen. This is easy to determine on an OCT scan by the intra-retinal or sub-retinal position of the hyper-reflective lesion. OCT negative scans without lesions confirming the status as M1 were sent back for re-grading of the original image (see Fig. 1 for clinical pathway for R1/M1 patients).

The main outcome measure was the number of SDOCT positive, borderline and negative episodes. We also detected other (non-diabetic) pathologies.

Retinopathy screening images were obtained according to UK National Screening Committee guidelines and OCT images were graded within the ophthalmology clinic. All analyses and data transfer were performed without any associated patient specific identifiers with the approval of the Local Ethics Committee and Caldicott Guardian. As such written consent is not required for patients' information to be stored and used for research.

## Results

There were 311 patients with diabetes and a diagnosis of R1/M1 on graded fundus images in either eye. These patients underwent SDOCT imaging of the maculae of both eyes between March 2008 and Sept 2009.The outcomes from the data collected at SDOCT clinic visit (OCT scan, visual acuity and colour fundus photography) are summarised in Table 2. The number of patients who went through the patient pathway is included in Figure 1.Of these, there were 7 patients with one or more un-assessable images (technical failures, 2.3% of the total audit) that required referral to an ophthalmology clinic. One hundred and nineteen screening episodes (38.3% of the total) were SDOCT maculopathy positive and referred to the medical retina clinic. One hundred and thirty one SDOCT negative (42.1%) and 12 SDOCT borderline episodes (3.9%) were given further appointments in the OCT clinic. Twenty-four patients (7.7%) were referred back to screening for a variety of reasons (Table 2). Twenty-five patients (8.0%) were referred to ophthalmology for reasons other than technical failure (Table 2).

**Table 2:** Outcome of primary SDOCT visits (n = 311).

Outcome	Reason	No
Referred for maculopathy	Positive OCT	119
Referred for other than maculopathy (n = 25)	Technical failure	7
	Epiretinal membrane/traction	5
	Age related macular degeneration	2
	Branch retinal vein occlusion	1
	Cataract	1
	Macular telangiectasia	1
	Central serous retinopathy	1
	Refraction and primary care	1
	Other: Hard drusen; choroidal folds, macular crystals; unexplained poor vision, peripapillary lesion; RAP; pigment epithelial detachment	6
Further OCT (n = 143)	Negative OCT	131
	Borderline OCT	12
Referred back to screening programme (n = 24)	Improved/unreliable VA	8
	Resolved maculopathy	5
	Drusen ± RPE changes	4
	Exudates >1 DD from fovea	4
	Macula crystal	1
	Other: refraction; artefact;	2

RAP; retinal angiomatous proliferation, RPE; retinal pigment epithelium, DD; disc diameter.

Table footnote: SDOCT borderline scans had an intra-retinal cystic space on a single scan without a change in the ILM contour. This group has recently been published as having a variety of causes for this appearance, not limited to DME, and was identified as a group to be observed. [20]

## Discussion

In developed countries, diabetic eye disease is the leading cause of blindness in adults aged under 65 years.[33] Macular edema has traditionally been assessed by a combination of clinical examination, stereoscopic retinal photographs and fluorescein angiography, with considerable argument within the literature and within the diabetic retinopathy screening community as to which method represents the ‘gold standard’. In reality, all of these methods are somewhat subjective and rely on an en face determination of retinal thickening. OCT imaging creates a thickness profile of the retina that mimics quite accurately the histological arrangement of the retina and has the additional benefit of some measure of objectivity.

The presence of clinically significant macular edema (CSME) increases the risk of moderate visual loss to approximately 30% to 50%, depending on the level of baseline visual acuity. [34] Diabetic macular edema (DME) was defined on the basis of stereoscopic fundus photography in ETDRS studies.[4] This technique is complicated and difficult to use in a clinical setting and was replaced with contact fundus biomicroscopy, which was found to be in close agreement with stereophotography, particularly for CSME.[35] CSME was defined in the ETDRS [4] as any retinal thickening within 500 microns of the center of the macula, or hard exudates within 500 microns of the center of the macula with adjacent retinal thickening, or retinal thickening at least 1 disc area in size, any part of which is within 1 disc diameter of the center of the macula. Noncontact fundus biomicroscopy is more commonly used, but has been shown to be slightly less sensitive than contact fundus biomicroscopy.[16]

There is no requirement for a particular visual acuity level to diagnose CSME and therefore initiate treatment. This is a clinical diagnosis used by an ophthalmologist in order to determine a level of severity of edema that will require laser treatment in order to prevent future vision loss. This is significantly different from the definition of diabetic maculopathy used in photographic screening programmes. For example, in the English National Screening Programme (ENSP), if the only detectable abnormality within the macula on the photograph is an isolated microaneurysm then it only merits referral to an ophthalmologist when the visual acuity falls to 20/40. There are arguments that the ENSP definitions will not identify patients with macular edema and good visual acuity, but this applies to most large scale population screening programmes for diabetic retinopathy. The solution to this problem is to use stereoscopic fundus photography, which was considered by the ENSP not to be sufficiently cost-effective or reliable outside of the context of international grading centres and clinical trials.

A review of the literature regarding the reliability of a clinical diagnosis of DME shows that this can be a quite variable ‘standard’. The sensitivity and specificity of a clinical diagnosis of diabetic CSME on a dilated fundus examination with seven-field stereo fundus photographs at a reading centre is 24% and 98% respectively, with a positive predictive value 71%, and negative predictive value 89%.[36] Harding et al. reported a sensitivity of 64% (20/33) for direct ophthalmoscopy by an experienced ophthalmologist relative to biomicroscopy with 60- and 90-diopter lenses by a retina specialist, for detection of sight-threatening maculopathy, defined as hard exudates within 1 DD of fixation, a circinate ring of hard exudates at least one disc area in size greater than 1 DD from fixation or scars of focal/grid photocoagulation.[37] Using a similar definition in approximately 700 eyes, Lee et al. identified maculopathy about half as frequently with non-stereo 45° photography as with biomicroscopy using a 90- diopter lens (10.2% vs. 4.9%, kappa 0.44). [38] Another study reported sensitivity of 45% for macular edema detected by retina specialists using indirect ophthalmoscopy routinely and slit-lamp biomicroscopy relative to seven-field stereoscopic photographs.[39] In the ETDRS, for detection of CSME, examinations with slit-lamp biomicroscopy by retina specialists who had developed the definition of CSME had a sensitivity of 82% (533/650, kappa 0.61) relative to seven-field stereoscopic photographs.[35], [40] Another photographic method, fluorescein angiography, was utilized in the ETDRS studies, but angiographic features were not included in the diagnosis of CSME.

Spectral domain OCT demonstrates greater sensitivity than fluorescein angiography in diagnosing cystoid macular edema (CME). There were no cases of clinical diagnosis of CME that were missed by SDOCT in a recent study. [20] A further recent study systematically reviewed the performance of OCT as a potential objective and quantitative alternative to fundus photography as a gold standard. The expected operating point on the summary ROC (Receiver operating characteristic), a pooled estimate of all studies, corresponded to a sensitivity of 0.79 (95% CI: 0.71–0.86), a specificity of 0.88 (95% CI: 0.80–0.93), a positive likelihood ratio of 6.5 (95% CI: 4.0–10.7), and a negative likelihood ratio of 0.24 (95% CI: 0.17–0.32). These values suggest a good overall performance of OCT for diagnosing CSME.[10]

For these reasons, OCT measurement of DME is now utilized as a standard clinical trial endpoint for treatments of DME in large multi-centre trials.[21]–[24] In addition, there are now multiple studies demonstrating high levels of repeatability of macular thickness measurements made with OCT.[41]-[44] Although caution should be exercised in that measurements cannot be used interchangeably between OCT machines due to the underlying software paradigm used to generate the retinal thickness.[45]. The consistent and objective nature of OCT parameters has even lent itself to evaluation as an automated tool for the assessment of CSME. [46]

The role of OCT in systematic population screening of patients with diabetes for DME has not been determined. There are several large studies in process that may answer some of these questions.[47] The principal questions to answer will be whether OCT screening increases referral of patients with early disease who are not at risk of vision loss, what is normal macular thickness in patients with diabetes across a range of age groups, refractive errors and ethnicities, what OCT parameters most closely match the clinical diagnosis of CSME, and whether the significant current costs of OCT can be reduced by advances in technology and software, such as automation.

This audit does not aim to answer those questions, but to evaluate the presence of macular pathology suggesting vascular leakage on OCT imaging of patients with a photographic screening diagnosis of M1. We aimed to refine the diagnosis of M1 in favour of patients with maculopathy amenable to currently available therapies i.e. those for DME, such as argon retinal laser, intravitreal triamcinolone and intravitreal anti-VEGF therapies. At present, the OCT parameters attributable to macular edema are well defined – increased retinal thickness or intraretinal or subretinal hyporeflective spaces representing extracellular fluid. In our opinion, the OCT parameters that correlate with a clinical diagnosis of CSME are insufficiently defined or validated to be applied to a retinal screening programme. Our triage paradigm was that all patients with evidence of definite edema should have a clinical evaluation. It is possible that future studies may further refine the OCT analysis and allow only the referral of patients definitely requiring treatment.

There are interesting questions regarding whether neurodegeneration precedes the development of the vascular changes in diabetic retinopathy. [48]–[50] Arguments regarding the effect of retinal neurodegeneration caused by diabetes on retinal thickness parameters are scientifically interesting, but, in the absence of specific therapies aimed at this neurodegeneration, are of mostly academic interest. More specifically, a patient with an equal degree of retinal thinning due to neurodegeneration and retinal thickening due to vascular leakage would have undetectable changes on clinical examination or stereoscopic fundus imaging and would not be considered to meet the thresholds for initiation of therapy for macular edema.

This audit shows that, in an unselected screening population of 17, 551 patients with diabetes mellitus (type 1 and type 2) derived from an urban inner city environment (central London, England) 311 were evaluated by standard photographic grading as having mild to moderate non-proliferative diabetic retinopathy (R1) in association with photographic surrogate markers of diabetic maculopathy (M1). Of these, only 38.3% had OCT evidence of macular edema and required examination by an ophthalmologist to evaluate the need for treatment. We believe that OCT is a useful adjunct to traditional photographic retinal screening and warrants further evaluation, particularly with respect to the role of OCT in screening pathways and the health economics of this introduction.

This analysis shows that patients with diabetes, mild to moderate non-proliferative diabetic retinopathy (R1) and evidence of diabetic maculopathy on non-stereoscopic retinal photographs (M1) have a 42.1% chance of having no macular edema on SDOCT imaging as defined by standard OCT definitions of DME when graded by a retinal specialist. SDOCT imaging is a useful adjunct to colour fundus photography in screening for referable diabetic maculopathy in our screening population.
